# Identifying Risk Factors, Patient-Reported Experience and Outcome Measures, and Data Capture Tools for an Individualized Pain Prediction Tool in Pediatrics: Focus Group Study

**DOI:** 10.2196/42341

**Published:** 2022-11-15

**Authors:** Michael D Wood, Nicholas C West, Rama S Sreepada, Kent C Loftsgard, Luba Petersen, Julie M Robillard, Patricia Page, Randa Ridgway, Neil K Chadha, Elodie Portales-Casamar, Matthias Görges, Pediatric Pain Prediction Collaboration

**Affiliations:** 1 Department of Anesthesiology, Pharmacology & Therapeutics University of British Columbia Vancouver, BC Canada; 2 BC Children’s Hospital Research Institute Vancouver, BC Canada; 3 Patient Partner Vancouver, BC Canada; 4 Department of Economics Simon Fraser University Vancouver, BC Canada; 5 Division of Neurology Department of Medicine University of British Columbia Vancouver, BC Canada; 6 BC Children’s Hospital Vancouver, BC Canada; 7 Division of Otolaryngology-Head and Neck Surgery Department of Surgery BC Children’s Hospital Vancouver, BC Canada; 8 Department of Pediatrics University of British Columbia Vancouver, BC Canada; 9 See Acknowledgments

**Keywords:** patient-oriented research, patient-reported outcome measures, patient-reported experience measures, risk prediction, pain, individualized risk, surgery, anesthesia, focus groups, thematic analysis, perioperative, participatory medicine, digital health tool, postsurgical pain, children, opioid use, virtual focus group, postoperative, pediatrics, risk prediction, health outcome

## Abstract

**Background:**

The perioperative period is a data-rich environment with potential for innovation through digital health tools and predictive analytics to optimize patients’ health with targeted prehabilitation. Although some risk factors for postoperative pain following pediatric surgery are already known, the systematic use of preoperative information to guide personalized interventions is not yet widespread in clinical practice.

**Objective:**

Our long-term goal is to reduce the incidence of persistent postsurgical pain (PPSP) and long-term opioid use in children by developing personalized pain risk prediction models that can guide clinicians and families to identify targeted prehabilitation strategies. To develop such a system, our first objective was to identify risk factors, outcomes, and relevant experience measures, as well as data collection tools, for a future data collection and risk modeling study.

**Methods:**

This study used a patient-oriented research methodology, leveraging parental/caregiver and clinician expertise. We conducted virtual focus groups with participants recruited at a tertiary pediatric hospital; each session lasted approximately 1 hour and was composed of clinicians or family members (people with lived surgical experience and parents of children who had recently undergone a procedure requiring general anesthesia) or both. Data were analyzed thematically to identify potential risk factors for pain, as well as relevant patient-reported experience and outcome measures (PREMs and PROMs, respectively) that can be used to evaluate the progress of postoperative recovery at home. This guidance was combined with a targeted literature review to select tools to collect risk factor and outcome information for implementation in a future study.

**Results:**

In total, 22 participants (n=12, 55%, clinicians and n=10, 45%, family members) attended 10 focus group sessions; participants included 12 (55%) of 22 persons identifying as female, and 12 (55%) were under 50 years of age. Thematic analysis identified 5 key domains: (1) demographic risk factors, including both child and family characteristics; (2) psychosocial risk factors, including anxiety, depression, and medical phobias; (3) clinical risk factors, including length of hospital stay, procedure type, medications, and pre-existing conditions; (4) PREMs, including patient and family satisfaction with care; and (5) PROMs, including nausea and vomiting, functional recovery, and return to normal activities of daily living. Participants further suggested desirable functional requirements, including use of standardized and validated tools, and longitudinal data collection, as well as delivery modes, including electronic, parent proxy, and self-reporting, that can be used to capture these metrics, both in the hospital and following discharge. Established PREM/PROM questionnaires, pain-catastrophizing scales (PCSs), and substance use questionnaires for adolescents were subsequently selected for our proposed data collection platform.

**Conclusions:**

This study established 5 key data domains for identifying pain risk factors and evaluating postoperative recovery at home, as well as the functional requirements and delivery modes of selected tools with which to capture these metrics both in the hospital and after discharge. These tools have been implemented to generate data for the development of personalized pain risk prediction models.

## Introduction

### Background

Persistent postsurgical pain (PPSP) is common in children [1] and is associated with detrimental consequences [2-4]. Although up to half of the variance in PPSP is attributable to genetic factors [5], modifiable factors have also been identified, such as psychosocial factors, such as anxiety, poor pain-coping mechanisms, and pain catastrophizing [1,6-8]. Although these risk factors are known, these findings have not yet been widely translated into algorithmic decision-making to guide personalized interventions, which could improve clinical outcomes, such as acute postoperative pain.

The perioperative period is a data-rich environment with potential for innovation through digital health tools and predictive analytics [9,10]. One such domain ripe for transformational change is the opportunity to use the preoperative period to optimize a patient’s health by performing targeted prehabilitation, which is a process of improving the functional capability of a patient prior to surgery to withstand the surgical insult and facilitate a return to preoperative conditions. Prehabilitation programs for adults [11,12] focusing on healthy eating and nutritional supplementation [12,13], improving physical function and exercise capacity [12,14], providing psychosocial interventions [15,16], and presurgical opioid weaning [17] can ﻿reduce the length of hospital stay; postoperative complication rates, such as pneumonia or wound infection [18]; and postoperative pain [19]. In pediatrics, similar concepts are being introduced in children with muscular and neurologic disease undergoing surgery [20] and are being developed for use in children undergoing spinal surgery [21].

To help develop strategies to improve family-centered care, patient-reported outcome measures (PROMs) are being implemented as standardized and validated questionnaires to systematically quantify patient perceptions regarding their health status [22], such as pain/discomfort and mobility. Furthermore, patient-reported experience measures (PREMs) can be used to quantify patient opinions regarding their health care encounter [22]. PROMs and PREMs are fundamental to personalization of care and should be ideally suited to developing risk prediction models targeting family-relevant experiences and outcomes.

### Objectives

Our long-term goal is to reduce the incidence of PPSP, and consequently chronic long-term postoperative opioid use, by developing personalized pain risk prediction models that can guide clinicians and families in identifying and selecting prehabilitation strategies to reduce acute postoperative pain. To develop such a system, our first steps were (1) to use patient-oriented research principles [23] and clinical expertise to identify risk factors for pediatric postoperative pain, as well as identify the PROMs and PREMs that are most meaningful in evaluating postoperative recovery, and (2) to select the appropriate tool(s) to capture these metrics both in the hospital and after discharge so that we can collect data for future pain risk modeling.

## Methods

### Study Design

We conducted a semistructured qualitative study through focus groups with parents of children who had previously undergone surgery, adults with lived pediatric surgical experience, and clinicians who work at BC Children’s Hospital (BCCH) in Vancouver, BC, Canada.

### Ethical Considerations

Approval was obtained from the University of British Columbia/Children’s & Women’s Health Centre of British Columbia Research Ethics Board (H21-00658; date of approval July 12, 2021; principal investigator: author MG).

### Participant Recruitment and Eligibility

Allied health professionals at BC Children’s Hospital were approached via departmental email distribution lists. To ensure our sample was representative and included a range of surgical procedures, parents were recruited in person in 2 surgical clinics within BC Children’s Hospital (orthopedics and otorhinolaryngology), during their child’s hospital visit, or in the anesthetic care unit (ACU), which provides perioperative care for children of all ages undergoing a variety of elective surgical procedures. Adults with previous childhood surgery were recruited via provincial research networks (Reach BC and the BC Children’s Hospital patient experience office e-network). Informed consent was obtained by research staff in person or electronically using Research Electronic Data Capture (REDCap, Vanderbilt University) [24,25] hosted at the BC Children’s Hospital Research Institute. Due to the small sample size and its consequent privacy concerns, parents and participants with pediatric lived experience will not be differentiated and will be collectively referred to as family members, as guided by the advice of our research ethics board.

As the focus groups were conducted virtually, participants without an internet connection and access to an electronic device were ineligible for recruitment. To encourage participation, participants were remunerated CA $25 (approximately US $18.35) per session for their expertise and time. Mixed panels of approximately 2-3 family members and 2-3 clinicians were targeted for each focus group.

### Data Collection

A brief prestudy questionnaire was administered using REDCap to collect participants’ demographic information. Two research team members with expertise in qualitative methods conducted 10 virtual focus groups between October 2021 and April 2022 using Zoom (Zoom Video Communications): one researcher facilitated each session (authors MDW or RS), while another took notes (author MDW or RS or Kim Correa [KC]). At the start of each focus group, a brief overview of our research program was provided, and we indicated that our objective was to identify (1) preoperative variables that may be associated with pain following surgery as well as PREMs and PROMs to collect postoperatively and (2) potential tools/instruments that could be implemented for data collection in the hospital and following discharge. Two sessions were conducted: The first was focused on objective 1; these participants were later contacted to return for a second session, in which we reviewed the major findings from the previous session and discussed objective 2.

In session 1, an open-ended discussion was structured around 4 themes: (i) *presurgical* variables that might be relevant to poor surgical outcomes; (ii) whether each of the identified presurgical variables related specifically to the patient, the parent/caregivers, or both; (iii) *postsurgical* PREMs and PROMs that represent a meaningful evaluation of the recovery process; and (iv) a discussion of additional relevant features of the perioperative and recovery periods.

In session 2, 3 themes were discussed: (i) potential instruments (if known) that could be used for data collection, (ii) potential functional requirements and delivery mode considerations for surveys to capture these data, and (iii) how to achieve effective implementation of these data collection tools both in the hospital and after discharge.

Each session lasted approximately 1 hour, was audio-recorded, and was digitally transcribed using the live transcription function in Zoom. Transcripts were verified by a member of the research team (KC) and participant names replaced by sequential identifiers.

### Data Analysis

Focus group transcripts were analyzed using NVivo (QSR International), and results were summarized using thematic analysis [26]. Two research team members (MDW and KC) independently reviewed two transcripts and used inductive coding to organize the data by theme, subtheme, and participant type [27]. These researchers then compared interpretations and developed consistent codes, which were applied to the remaining 4 transcripts (deductive coding); the 2 researchers discussed additional themes that emerged, resolved any further discrepancies, and modified the coding framework iteratively to ensure that key concepts were not overlooked and that the coding framework remained consistent. Due to the qualitative nature of the study, we did not estimate a target sample size and instead applied a saturation criterion, which indicated that once similar comments and concerns were repeatedly discussed across focus groups, saturation had occurred, and participant recruitment could conclude.

### Tool Selection for Future Data Capture

Following the focus group thematic analysis, the research team used a combination of these findings and targeted literature reviews to identify specific data capture tools and questionnaires that satisfied the key requirements arising from the focus group discussions. In brief, we searched the literature, using the terms given by our participants, as well as using related terms or synonyms, to identify tools that (1) most closely matched our participants’ meaning, (2) were feasible to implement, and (3) had been validated in a similar population or setting. This selection was further guided by multiple team meetings to gain expert consensus among researchers, clinicians, and patient partners. Finally, tools for implementation in a future data collection and risk modeling study were proposed.

## Results

### Focus Group Participant Demographics

In total, 22 participants were included. Participant demographics were as follows: 12 (55%) clinicians (n=2, 17%, registered nurses, n=2, 17%, nurse practitioners, n=1, 8%, surgeon, and n=7, 42%, anesthesiologists) and 10 family members attended 10 focus group sessions: 2 (20%) of the 10 sessions included 2 participants per session, and the 8 (80%) remaining sessions included 3-4 participants per session; 4 (40%) of the 10 sessions were mixed groups (combining clinicians and family members). When approached in the clinic, 5 family members declined due to a lack of interest and 2 clinicians declined due to limited availability; 2 family members declined to participate following informed consent due to limited availability. Participants included 12 (55%) of 22 persons identifying as female, and 12 (55%) were under 50 years of age. Clinicians worked in surgery, anesthesiology, and pain management (n=8, 67%) and perioperative/perianesthesia nursing (n=4, 33%). Family member participants included 9 (90%) of 10 with either a certificate (university/nonuniversity) or a university degree and 1 (10%) with a high school diploma (or equivalent).

### Key Domains for Data Capture

Comments from focus group participants were grouped into 5 domains, described in the following sections, with a list of quantifiable metrics summarized in Table 1.

**Table 1:** Key metrics identified from focus groups with clinicians, allied health professionals, and family members to be used for future data collection.

Domain	Metrics to capture
Demographic risk factors	Child factors: age, sex at birth, weight
	Family factors: level of education, household income, ethnicity, primary language
Psychosocial risk factors	Anxiety; (pain) catastrophizing, depression, medical phobia(s), obsessive compulsive disorder, posttraumatic stress disorder, coping strategies (stressful situations), support network availability
Clinical risk factors	Type of surgery, number of previous surgeries, pre-existing conditions (eg, chronic pain and previous response to anesthetics), history of narcotic/analgesic use or abuse, administered medications (eg, multimodal pain management) during the perioperative period
PREMs^a^	Coordination of care, access to care, clarity of discharge instructions, satisfaction with care
PROMs^b^	Functional recovery: eating and drinking, nausea or vomiting, bowel movements and urination, mobility, return to school and play activities, length of hospital stay, prescribed medications at hospital discharge Undesired postprocedural side effects: surgical site infection, bleeding, pain severity and duration, number of readmissions or seeking of urgent care

^a^PREM: patient-reported experience measure.

^b^PROM: patient-reported outcome measure.

#### Demographic Variables

Clinicians noted that adolescents tend to experience increased pain following surgery, whereas younger children recover more quickly; families largely agreed but also indicated that younger children are “a lot more nervous” about undergoing surgery (family member 1), whereas teenagers have “more control over the situation and decision-making power” (family member 2) to decrease potential anxiety. The child with an increased BMI may be “underdosed on pain medications” (clinician 1), and optimizing a “patient’s nutrition and fitness level” (clinician 2) may improve outcomes. Some clinicians recommended capturing socioeconomic aspects that may impact the child’s recovery (clinician 1) and indicated that language barriers may cause “difficulties for health care professionals to explain and set realistic expectations for families and prepare them for the postoperative period” (clinician 3).

#### Psychosocial Factors

Most clinicians and family members indicated that we should quantify both parent and patient anxiety. Increased anticipation of surgery-induced pain may lead to catastrophizing, where the parent has the potential to “excessively fuel the emotional state of the child” (family member 3). Children “can sense the anxiety and the changes in behavior of their parents,” which may increase postoperative complications when compared to children observing “parents that are calm and understanding” (clinician 3). Due to their association with anxiety, capturing information about depression, medical phobias (specifically needle phobia), obsessive compulsive disorder, and posttraumatic stress disorder was also suggested. Some family members and clinicians believed that patients who do not cope well with “stress or with new situations” (clinician 7) may struggle following surgery. Finally, families and clinicians indicated that assessing the availability for a family’s support network following discharge may also be imperative to ensuring optimal recovery.

#### Clinical Characteristics

Clinicians noted that the type of surgery is associated with varying levels of expected postoperative pain, depending on extent and location, with multiple surgeries potentially “leading to chronic pain syndromes” (clinician 5), which may increase pain following surgery. The patients’ pre-existing conditions, including chronic or prolonged pain following a previous surgery, or an atypical response to anesthetics or a history of opioid analgesic use or substance abuse “may [also] affect the amount of analgesia that is required to achieve [optimal] pain control” (clinician 6). Clinicians further indicated that the class and dose of medications administered during the perioperative period, as well as any multimodal pain management, will be imperative to capture due to their beneficial effect in managing intra- and postoperative pain. Finally, clinicians suggested that we quantify the planned length of hospital stay as a surrogate for medical complexity, as well as unplanned readmission(s) or seeking urgent care.

#### Patient-Reported Experience Measures

Some family members indicated that poor coordination of postoperative care results in “a distinct contrast in the experiences of people who are [connected with] primary care for follow-up compared to those who are not” (family member 3). Several participants suggested that a negative experience with health care can create stress and adversely impact both recovery and the attitudes toward subsequent medical procedures. Furthermore, although care may be easily acquired within the hospital, access becomes more difficult once discharged back into the family’s community. Family members further indicated that discharge instructions are meant to educate and set realistic expectations, but worried that ambiguity could produce “a lack of confidence” (family member 4) and may compromise effective pain management.

#### Patient-Reported Outcome Measures

Clinicians and family members primarily indicated the importance of returning to normal physical function, such as capturing whether patients are eating and drinking, vomiting or feeling nauseated, having “normal” bowel movements and urination, or experiencing undesired procedural side effects, such as surgical site infection(s) or postoperative bleeding. In addition, family members believed it would be imperative to ask questions such as “Are you playing?” (family member 4), “Are you able to go to school?” (family member 4), and “Are you capable of managing stairs?” (family member 1), which represent common activities of daily living and functional recovery for children and adolescents. Finally, participants believed that we should continually capture “the severity, duration, and trajectory of postoperative pain” with “developmentally appropriate [pain scales]” (clinician 4).

### Requirements and Modes for Data Capture Tools

The second iteration of focus groups identified key functional requirements and delivery modes considerations for future data capture tools and questionnaires (Table 2).

**Table 2:** Key functional requirements and delivery modes for data collection tools, identified from the second iteration of focus groups with clinicians and family members.

Domain	Considerations
Functional requirements	Using primarily standardized and validated scale-based tools, including Likert scales and multiple-choice questions Sparingly using open-ended questions (to ensure the patient’s voice is heard) Collecting repeated measurements (eg, postoperative days 1, 2, 3, 7) Ensuring brief survey completion times (no longer than 15 minutes) Using binary questions and branching logic to create dynamic surveys asking only relevant questions Providing save (and resume) functionalities Enabling notifications and reminders Having an opt-out option
Delivery modes	Primarily electronic Providing an alternate paper-and-pencil or telephone option Having both parent proxy and self-report versions
Compensation requirement	Remunerating participants for their time, expertise, and lived experience

#### Functional Requirements

Clinicians and family members indicated that standardized and validated scale-based tools should be implemented to streamline administration of multiple surveys and optimize comparability across studies and hospitals. Participants further indicated that surveys should be “quick and easy,” increase “accessibility,” and ensure “a representative sample” (clinician 9), for example, Likert scales and multiple-choice questions. Open-ended questions should be used sparingly but should be included to ensure patients “have their voice heard” (family member 6). Clinicians and family members further suggested repeating surveys, such as postoperative days 1, 2, 3, and 7, which should take less than 10-15 minutes each and should stop at 3 months, as survey fatigue/attrition may be a potential barrier. Binary “yes/no” questions and branching logic could substantially reduce survey completion times with future questions, depending on choices made by participants. Some family members and clinicians indicated that the survey should be savable (and resumable) and include notifications to ensure survey completion. Family members also suggested that having an opt-out feature would allow participants an opportunity to stop participating at any time. Finally, participants indicated that patients should receive monetary incentives to encourage survey completion and reward participants for their time.

#### Delivery Modes

Most participants believed that we should “collect information as seamlessly as possible” (family member 5), principally via electronic survey administration. However, both family members and clinicians also indicated that we should “provide multiple options” (clinician 9) so families can choose their preferred format, whether paper and pencil, electronic, or a telephone call, to go through the survey with a research team member and ask clarification questions. Some participants indicated that parent proxy surveys should be used to assess children under 13 years of age, whereas adolescents could self-report questionnaires with parental/guardian consent. Parents largely agreed and further indicated that self-report measures would be valuable to assist in understanding the child’s perspective as “they're not always forthcoming to their parents” (family member 5).

### Design of a Perioperative Data Collection Platform

**Figure 1:**
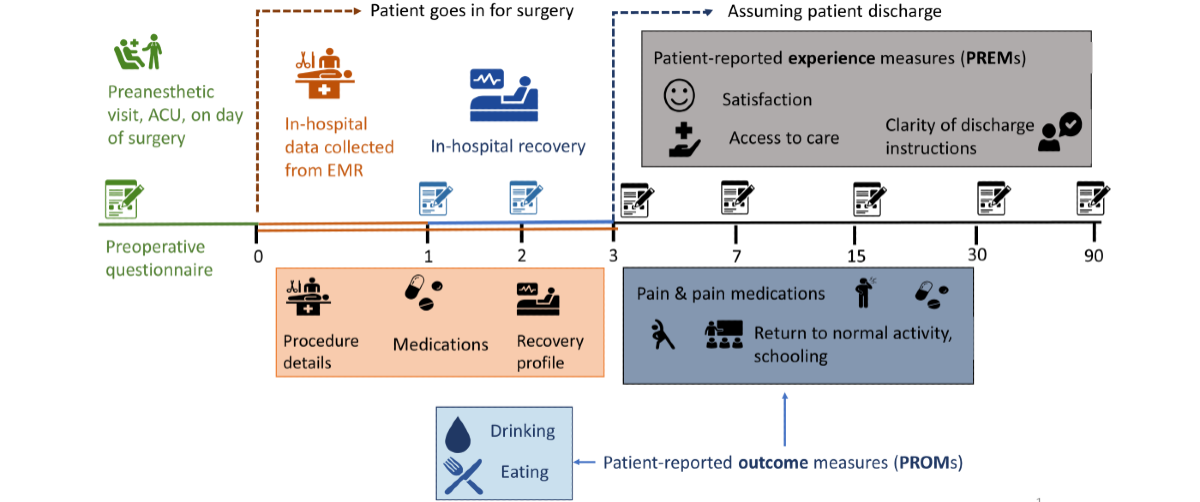
Proposed perioperative data collection timeline. ACU: anesthetic care unit; EMR: electronic medical record.

Based on the proposed key metrics, the functional requirements, and delivery modes identified by participants, as well as a brief literature review, we designed a perioperative data collection platform to gather preoperative risk factors, intraoperative and in-patient data for hospitalized patients, and postoperative PREMs/PROMs (Figure 1).

PROMs will be collected using the Patient-Reported Outcomes Measurement Information System (PROMIS) [28] due the extensive library of standardized, validated, and Likert scale–based questionnaires, brief administration times, and the ability to evaluate and monitor multiple patient-oriented domains in parallel via repeated application (on postoperative days 1, 2, 3, 7, 15, 30, and 90). This will also allow for multiple data capture methods (electronic, telephone, or paper and pencil) as well as different modes of administration, such as self-reporting (patient age>12 years) and parent proxy (patient age≤12 years).

#### Tools for Data Capture

To decrease potential redundancy, patient factors, such as age and sex at birth, will be extracted from electronic medical records (EMRs), whereas family factors, such as household income and ethnicity, are appropriate for self-reporting. PROMIS anxiety, depression, and social relationship tools will capture patients’ preoperative psychosocial data; parental pain catastrophizing will be captured using the pain-catastrophizing scale (PCS) [29] or its child version (PCS-C) [30] for adolescent self-reporting. The Car, Relax, Alone, Forget, Friends, Trouble (CRAFFT) [31] questionnaire will be used to *optionally* self-report the adolescents’ history with substance abuse. The in-hospital data, including the type of surgery, length of hospital stay, and administered medications, can be collected from the EMRs, whereas pain intensity, pain interference, and physical function need to be captured via PROMIS tools as they continue to be collected past hospital discharge. Additionally, PREMs will be captured via parent proxy on postoperative days 15, 30, and 90 based on the pediatric care transitions measure [32], whereas PROMs will be gathered on postoperative days 1, 2, 3, 7, 15, 30, and 90 using PROMIS [28] tools. Finally, in-hospital outcomes, including nausea, vomiting, and pain, will be transcribed from EMRs and subsequently captured via parent proxy reporting following discharge.

## Discussion

### Principal Findings

Using the preoperative period to optimize patients’ health by performing targeted prehabilitation is an opportunity to improve patient outcomes; specifically, digital health innovations might transform how perioperative care can be delivered in a personalized and family-centered way. Thematic analysis of focus groups identified 5 key domains for data capture to be used in risk modeling and to capture family-meaningful variables for pediatric postoperative recovery: (1) demographics, including age, sex, and weight; (2) psychosocial factors, including anxiety, depression, and medical phobias; (3) clinical characteristics, including pre-existing conditions, procedure type, and length of hospital stay; (4) PREMs, including patient and family satisfaction with care; and (5) PROMs, including nausea and vomiting, functional recovery, and return to normal activities of daily living. Participants further identified functional requirements, including the use of standardized and validated instruments, and repeated measures, to guide the selection of appropriate tools to capture these metrics, both in the hospital and following discharge, as well as specifying that data collection should be primarily electronic, with a paper-and-pencil option. The combination of well-established PREM/PROM questionnaires, PCSs, and substance use questionnaires for adolescents embodied these functional requirements in our proposed data collection platform.

### Comparison With Prior Work

Studies have demonstrated that pediatric patients frequently develop PPSP, which may include patterns of persistent opioid use following surgery [1,4,33,34]. A wide range of risk factors have been identified, including some that may be amenable to preoperative [4,35-39] or perioperative [33] mitigation; these are broadly in line with the findings of this study and include pre-existing chronic pain and presurgical pain intensity, pain coping, child anxiety, preoperative opioid/substance use, depression, and poor sleep quality. There is, in the pediatric domain, also significant evidence that parental anxiety can impact a child’s postoperative pain experience [40,41]. Although these risk factors can be modeled [42-44], a recent systematic review and meta-analysis indicated that there are few pediatric studies that have been conducted to model presurgical risk factors associated with PPSP [1]. Interestingly, preoperative demographic factors, including age, sex, and the BMI were not associated with PPSP, and preoperative pain intensity had only an inconsistent association [1]. In contrast, preoperative psychosocial factors were consistently associated with PPSP, including child anxiety, decreased efficacy of pain coping, and parental pain catastrophizing [1], which further highlights the importance of including these factors in predictive PPSP models.

Due to PPSP risk factors being well understood in adults, personalized predictive analytic frameworks and decision support tools have been developed and implemented in the adult perioperative domain [45-49]. Despite the potential value and benefits of listening to families when co-developing an eHealth tool, such as determining end-user preferences for content and feature considerations to effectively support patient self-management [50] and obtaining parents’ feedback to efficiently design clinical trials in young children [51], patient-oriented research principles and methods [52,53] are rarely used to guide data collection platform development and results in PREMs and PROMs are not commonly included in predictive algorithms [45-49]. As our hospital has established that parents are keen to play a role in research across the pediatric care spectrum [54], conducting patient-oriented research [23] is paramount to addressing this substantial opportunity for improvement.

The Personalized Risk Evaluation and Decision Making in Preoperative Clinical Assessment app is 1 of the few platforms that involved patient partners with lived surgical experience in the initial development [55]. Although the study collected some PREM and PROM data, including satisfaction, length of hospital stay, and anxiety, these metrics were not used to predict long-term outcomes following discharge [55]. Furthermore, the failure to include comprehensive family-relevant long-term outcomes in national registries, such as the National Surgical Quality Improvement Program [56], results in risk prediction models derived from these data sets potentially lacking long-term patient-oriented outcomes. Our findings highlight the feasibility of including patient-oriented research principles, as well as PREMs and PROMs, in predictive modeling in an effort to improve long-term outcomes following surgery.

### Limitations

First, our sample comprised a relatively small cohort of clinicians and family members, and only a small subset of our group provided specific feedback on our data collection instruments, both of which may limit the transferability (or generalizability) of our findings. Although our cohort did comprise a diverse cohort of health care team members and parents of children from multiple clinics in our hospital, and we extended their contributions with further review of the literature, broader engagement with patient representation organizations might be desirable in future work. Additionally, our findings may be biased due to the research team being affiliated with the same organization (BC Children’s Hospital) where the patients and family members are being recruited and receiving care, instead of being recruited using a multi-institutional approach. Second, it would be ideal to add children and adolescents to future sessions and to potentially develop young persons’ advisory groups [57] to ensure that risk factors, PREMs, and PROMs represent pediatric patient needs. Third, our focus groups comprised only English-speaking participants, which may also have limited transferability; language interpretation services and closed captioning were offered during recruitment, but the primary language may still have represented an obstacle to accessibility. Finally, capturing nonverbal data, such as kinetics, facial expressions, proxemics, and paralinguistics, was beyond the scope of this study but should be considered in future work, particularly if analyzing clinical, patient, or family requirements directly in a health care setting, or during evaluation of proposed solutions.

### Conclusion

Our study identified key domains in which to capture data for targeting postoperative pain risk factors, such as demographics, psychosocial factors, clinical characteristics, and family-relevant outcomes during the recovery period, such as PREMs and PROMs. Clinician and family participants indicated functional requirements and preferred delivery modes; combined with a targeted literature review, these requirements allowed us to find tools with which to capture the identified metrics both in the hospital and after discharge. These tools will be implemented to generate data to inform the development of personalized pain risk stratification models.

## Abbreviations

EMR: electronic medical record
PCS: pain-catastrophizing scale
PPSP: persistent postsurgical pain
PREM: patient-reported experience measure
PROM: patient-reported outcome measure
PROMIS: Patient-Reported Outcomes Measurement Information System
REDCap: Research Electronic Data Capture
